# Timing of radiotherapy (RT) after radical prostatectomy (RP): long-term outcomes in the RADICALS-RT trial (NCT00541047)

**DOI:** 10.1016/j.annonc.2024.03.010

**Published:** 2024-04-05

**Authors:** C. C. Parker, P. M. Petersen, A. D. Cook, N. W. Clarke, C. Catton, W. R. Cross, H. Kynaston, W. R. Parulekar, R. A. Persad, F. Saad, L. Bower, G. C. Durkan, J. Logue, C. Maniatis, D. Noor, H. Payne, J. Anderson, A. K. Bahl, F. Bashir, D. M. Bottomley, K. Brasso, L. Capaldi, C. Chung, P.W. Cooke, J. F. Donohue, B. Eddy, C. M. Heath, A. Henderson, A. Henry, R. Jaganathan, H. Jakobsen, N. D. James, J. Joseph, K. Lees, J. Lester, H. Lindberg, A. Makar, S. L. Morris, N. Oommen, P. Ostler, L. Owen, P. Patel, A. Pope, R. Popert, R. Raman, V. Ramani, A. Røder, I. Sayers, M. Simms, V. Srinivasan, S. Sundaram, K. L. Tarver, A. Tran, P. Wells, J. Wilson, A. M. Zarkar, M. K. B. Parmar, M. R. Sydes, the RADICALS investigators

**Affiliations:** 1 Institute of Cancer Research, Royal Marsden NHS Foundation Trust, Sutton, UK; 2 Department of Oncology, Copenhagen Prostate Cancer Center, Copenhagen University Hospital, Rigshospitalet, Copenhagen, Denmark; 3 MRC Clinical Trials Unit at UCL, Institute of Clinical Trials and Methodology, UCL, London; 4 Department of Urology, The Christie NHS Foundation Trust, Manchester; 5 Manchester Cancer Research Centre, The University of Manchester, Manchester; 6 Department of Urology, Salford Royal NHS Foundation Trust, Manchester, UK; Department of Urology, Wythenshawe Hospital, Manchester University NHS Foundation Trust, Manchester, UK; 7 Department of Radiation Oncology, Princess Margaret Cancer Centre, University Health Network, Toronto, Canada; 8 Department of Urology, St James’s University Hospital, Leeds; 9 Division of Cancer and Genetics, Cardiff University, Cardiff, UK; 10 Canadian Cancer Trials Group, Queen’s University, Kingston, Canada; 11 Department of Urology, Bristol Urological Institute, Bristol, UK; 12 Department of Urology, Centre Hospitalier de l’Université de Montréal, Montreal, Canada; 13 Guy’s and St Thomas’ NHS Foundation Trust, London; 14 Institute of Cancer Research, Royal Marsden NHS Foundation Trust, London, UK; 15 Department of Urology, University Hospital Galway, Galway, Ireland; 16 Department of Oncology, The Christie Hospital NHS FT, Wilmslow Road, Manchester; 17 The Prostate Centre, London; 18 St James’s Institute of Oncology, Leeds; 19 Bristol Haematology and Oncology Centre, University Hospitals Bristol & Weston NHS Trust, Bristol; 20 Queen’s Centre for Oncology, Castle Hill Hospital, Hull University Teaching Hospitals NHS Trust, Cottingham, UK; 21 Department of Urology, Copenhagen Prostate Cancer Center, Copenhagen University Hospital, Rigshospitalet, Copenhagen; 22 Department of Clinical Medicine, University of Copenhagen, Copenhagen, Denmark; 23 Worcester Oncology Centre, Worcestershire Acute NHS Hospitals Trust, Worcester; 24 Department of Urology, The Royal Wolverhampton NHS Trust, Wolverhampton; 25 Department of Urology, Maidstone and Tunbridge Wells NHS Trust, Maidstone; 26 East Kent University Hospitals Foundation Trust, Kent; 27 Department of Clinical Oncology, University Hospital Southampton NHS Foundation Trust, Southampton; 28 Leeds Institute of Medical Research, University of Leeds, Leeds; 29 Department of Urology, University Hospitals Birmingham NHS Foundation Trust, Birmingham, UK; 30 Department of Urology, Herlev University Hospital, Herlev, Denmark; 31 Leeds Teaching Hospitals; 32 York and Scarborough Teaching Hospitals, York; 33 Kent Oncology Centre, Maidstone and Tunbridge Wells NHS Trust, Maidstone; 34 South West Wales Cancer Centre, Singleton Hospital, Swansea, UK; 35 Department of Oncology, Herlev University Hospital, Herlev, Denmark; 36 Department of Urology, Worcestershire Acute Hospitals Trust, Worcester; 37 Wrexham Maelor Hospital, Wrexham; 38 Department of Urology, Hillingdon Hospitals NHS Foundation Trust, Hillingdon, London; 39 Bradford Royal Infirmary, Bradford; 40 Leeds Cancer Centre, Leeds; 41 Department of Urology, University College London Hospitals, London; 42 Kent Oncology Centre, Kent & Canterbury Hospital, Canterbury; 43 Deanesly Centre, New Cross Hospital, Wolverhampton; 44 Department of Urology, Hull University Hospitals NHS Trust, Hull; 45 Glan Clwyd Hospital, Betsi Cadwaladr University Health Board, Rhyl; 46 Department of Urology, Mid Yorkshire Teaching Hospital, Wakefield; 47 Department of Oncology, Queen’s Hospital, Romford; 48 Barts Cancer Centre, St Bartholomews Hospital, London; 49 Royal Gwent Hospital, Newport; 50 Department of Oncology, University Hospitals Birmingham, Birmingham, UK

**Keywords:** prostate cancer, radiotherapy, randomised controlled trial, clinical trial, observational, long-term follow-up

## Abstract

**Background:**

The optimal timing of radiotherapy (RT) after radical prostatectomy for prostate cancer has been uncertain. RADICALS-RT compared efficacy and safety of adjuvant RT versus an observation policy with salvage RT for prostate-specific antigen (PSA) failure.

**Patients and methods:**

RADICALS-RT was a randomised controlled trial enrolling patients with ≥1 risk factor (pT3/4, Gleason 7-10, positive margins, preoperative PSA≥10 ng/ml) for recurrence after radical prostatectomy. Patients were randomised 1:1 to adjuvant RT (‘Adjuvant-RT’) or an observation policy with salvage RT for PSA failure (‘Salvage-RT’) defined as PSA≥0.1 ng/ml or three consecutive rises. Stratification factors were Gleason score, margin status, planned RT schedule (52.5 Gy/20 fractions or 66 Gy/33 fractions) and treatment centre. The primary outcome measure was freedom-from-distant-metastasis (FFDM), designed with 80% power to detect an improvement from 90% with Salvage-RT (control) to 95% at 10 years with Adjuvant-RT. Secondary outcome measures were biochemical progression-free survival, freedom from non-protocol hormone therapy, safety and patient-reported outcomes. Standard survival analysis methods were used; hazard ratio (HR)<1 favours Adjuvant-RT.

**Results:**

Between October 2007 and December 2016, 1396 participants from UK, Denmark, Canada and Ireland were randomised: 699 Salvage-RT, 697 Adjuvant-RT. Allocated groups were balanced with a median age of 65 years. Ninety-three percent (649/697) Adjuvant-RT reported RT within 6 months after randomisation; 39% (270/699) Salvage-RT reported RT during follow-up. Median follow-up was 7.8 years. With 80 distant metastasis events, 10-year FFDM was 93% for Adjuvant-RT and 90% for Salvage-RT: HR=0.68 [95% confidence interval (CI) 0.43-1.07, *P*=0.095]. Of 109 deaths, 17 were due to prostate cancer. Overall survival was not improved (HR=0.980, 95% CI 0.667-1.440, *P*=0.917). Adjuvant-RT reported worse urinary and faecal incontinence 1 year after randomisation (*P*=0.001); faecal incontinence remained significant after 10 years (*P*=0.017).

**Conclusion:**

Long-term results from RADICALS-RT confirm adjuvant RT after radical prostatectomy increases the risk of urinary and bowel morbidity, but does not meaningfully improve disease control. An observation policy with salvage RT for PSA failure should be the current standard after radical prostatectomy.

**Trial identification:**

RADICALS, RADICALS-RT, ISRCTN40814031, NCT00541047.

## Introduction

Radical prostatectomy is a standard treatment for localised prostate cancer, and may be followed by post-operative radiotherapy (RT) to the prostate bed.^1,2^ There has been uncertainty about the optimal timing of RT after radical prostatectomy. Adjuvant RT may be given early to those with no evidence of residual disease after surgery in order to reduce the risk of subsequent recurrence. Alternatively, salvage RT may be given later in the event of recurrent disease. It is possible that adjuvant RT might be more effective than a policy of salvage RT for recurrence. However, the salvage policy avoids unnecessary treatment of those cured by surgery alone and so may lead to less treatment-related morbidity.

Two randomised trials of adjuvant RT after radical prostatectomy were initiated over 30 years ago: The SWOG 8794 trial^3^ reported an overall survival benefit for adjuvant RT, but this survival benefit was not confirmed by the EORTC 22911 trial,^4,5^ and expert opinion was divided; at the Advanced Prostate Cancer Consensus Conference (APCCC) 2017, faced with a range of clinical scenarios, 48% of the panel voted in favour of adjuvant RT and 52% did not.^6^

There have been a further five randomised controlled trials comparing adjuvant RT versus a policy of salvage RT for recurrence. Until now, these trials have not been sufficiently large or mature enough to report on long-term outcomes such as overall survival or freedom-from-distant-metastasis (FFDM). These trials have reported an earlier outcome measure, biochemical progression-free survival (bPFS). The ARO 96-02 trial^7^ and the Finnish Radiation Oncology Group trial^8^ found that adjuvant RT reduced the risk of biochemical progression. However, prostate-specific antigen (PSA) failure at any time was regarded as an event, even in participants who subsequently went on to receive successful salvage RT. Therefore, a benefit in biochemical progression using this definition demonstrates that RT has activity but does not shed any light on its optimum timing. The remaining three randomised trials, RADICALS-RT,^9^ RAVES^10^ and GETUG-16,^11^ used a different definition of biochemical progression, requiring PSA failure after RT. This approach was designed to avoid the limitations of the previous definition but may have introduced a bias in favour of the salvage policy. A meta-analysis of these three trials found no bPFS benefit for adjuvant RT.^12^ Given the lack of robust early surrogate outcome measures, there remains a need to determine the effect of adjuvant RT on long-term outcomes such as FFDM and overall survival.

RADICALS-RT was designed to compare the efficacy and safety of adjuvant RT after radical prostatectomy versus a policy of observation with early salvage RT for PSA failure. With the benefit of longer-term follow-up, we now report on the primary outcome measure of FFDM.

## Patients And Methods

### Study design and participants

RADICALS is an international, phase III, multi-centre, open-label, randomised controlled trial in prostate cancer. The protocol contains two separate randomisations with over-lapping patient groups and was implemented at 138 trial-accredited centres in Canada, Denmark, Ireland and the UK. Participants were randomised shortly after radical prostatectomy between adjuvant and salvage post-operative RT (RADICALS-RT).

Patients with non-metastatic adenocarcinoma of the prostate were eligible for RADICALS-RT if they had undergone radical prostatectomy, had post-operative PSA≤0.2 ng/ml and at least one risk factor from the following: pathological T-stage 3 or 4; Gleason score 7-10; positive margins or preoperative PSA≥10 ng/ml. Appropriate ethical review was in place for each participating country. All participants gave written, informed consent.

### Randomisation

Participants were randomised within 22 weeks after radical prostatectomy to receive either adjuvant RT to the prostate bed±pelvis (‘Adjuvant-RT Group’), or close observation with salvage RT to the prostate bed±pelvis given in the event of PSA failure, defined as either: (i) two consecutive rising PSA levels with a PSA of >0.1 ng/ml, or (ii) three consecutive rising PSA levels (‘Salvage-RT Policy Group’). Randomisation using a 1:1 allocation was carried out centrally using minimisation with a random element which was stratified by the Gleason sum score, margin status, RT schedule and study centre.

### Treatment

RT to the prostate bed used a non-randomised dose-fractionation schedule of either 66 Gy in 33 fractions or 52.5 Gy in 20 fractions. Treatment commenced within 2 months of randomisation and within 26 weeks of radical prostatectomy for Adjuvant-RT, and within 2 months after PSA failure for Salvage-RT. RT could be delayed by up to 2 months further if the patient was also due to receive hormone therapy.

Participants could also receive RT to the pelvic lymph nodes at the investigator’s discretion. RT was planned with the patient supine, with empty rectum and comfortably full bladder. Patients could also receive up to 2 years of hormone therapy (either a luteinising hormone-releasing hormone (LHRH) analogue or bicalutamide 150 mg daily) starting before and continuing during and after their post-operative RT. The duration was either according to clinical judgement or by random allocation through participation in RADICALS-HD^13,14^ to either no, 6 months or 2 years duration of hormone therapy.

### Assessment for efficacy and adverse events

Patients were seen by a site investigator every 4 months from randomisation for 2 years, then 6-monthly until 5 years and then annually until 15 years. Clinician-reported data were collected at each follow-up visit on diarrhoea, proctitis, cystitis, haematuria and urethral stricture, graded according to the Radiation Therapy Oncology Group (RTOG) toxicity score.^15^ Data on other adverse events were collected if they met the criteria to be classified as a serious adverse event. Patient-reported data were collected at baseline, 1, 5 and 10 years after randomisation using standard questionnaires that included Vaizey (bowel) and the International Continence Society male short form (urinary incontinence).

### Outcome measures

The full design of RADICALS has been described previously.^16^ RADICALS-RT was designed to focus on long-term outcomes; the primary outcome measure was originally disease-specific survival, with FFDM as a key secondary outcome measure. Distant metastasis could be bone, liver, lung, distant node or other metastasis, but did not include pelvic nodes. With emerging data of improving patient outcomes from the EORTC 22911 and SWOG 8794 trials, and following discussion with the ongoing RAVES and GETUG-17 trials of RT timing, it was decided to change the primary outcome of the RADICALS-RT comparison to be FFDM, which would have greater power at any given time. This change was made with all ethical and regulatory approvals in place, without reference to accumulating comparative data from RADICALS-RT, and was agreed with the Trial Steering Committee (which includes independent members, including the chair) and gained favourable international peer review, through Cancer Research UK.

Secondary outcome measures included initiation of non-protocol hormone therapy, treatment toxicity and patient-reported outcomes. To facilitate the ARTISTIC meta-analysis, planned in collaboration with RAVES and GETUG-17, freedom from biochemical progression was added as a secondary outcome measure in 2018, again without reference to the accumulating, comparative data from RADICALS-RT and with the approval of the oversight committees.^12^ bPFS was defined as freedom from PSA≥0.4 ng/ml following post-operative RT, or PSA>2.0 ng/ml at any time, or clinical progression, initiation of non-protocol hormone therapy or death from any cause.

### Sample size

To target an improvement in participants free of distant metastasis at 10 years from 90% to 95%, with 80% power at a two-sided 5% significance level, would require 66 participants with distant metastasis events. This was anticipated to require 1063 participants at an accrual rate of 30 participants per month or 1160 participants at 25 participants per month.

### Statistical analysis

The analysis plan has been published.^17^ All analyses are carried out on an intention-to-treat basis. The statistical significance of differences between randomised groups were assessed using the log-rank test, and in the absence of evidence of non-proportional hazards, the hazard ratio (HR), from a Cox proportional hazards model, was reported as the measure of effect, with analyses stratified by the stratification factors used at randomisation (except centre). Toxicity data were divided into events reported as occurring within 2 years after randomisation and subsequently. Within each period, the highest grade of event experienced by participants was compared between randomised groups using the chi-square test. For patient-reported outcomes, groups are compared at 1, 5 and 10 years using analysis of covariance, adjusted for baseline score.

One sensitivity analysis was conducted, in which participants who had any metastatic event reported as ‘suspicious’ but which was not subsequently confirmed were assumed to have developed metastasis at that time.

Trial follow-up concluded on 31 December 2021 and the database was locked on 27 May 2022.

## Results

### Patients

**Figure 1:**
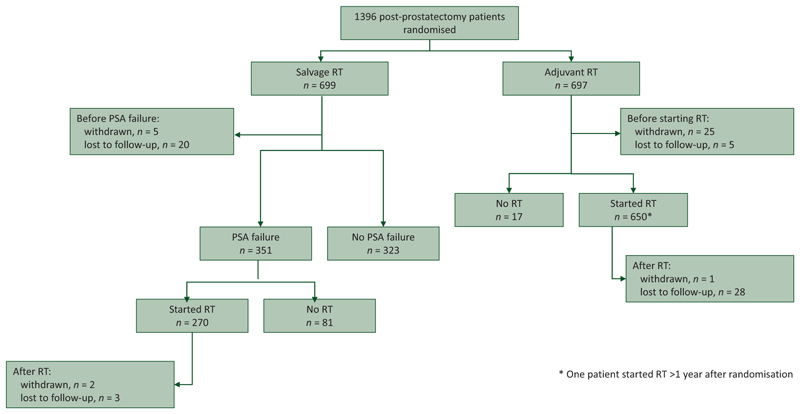
Accrual to RADICALS-RT and patient progress through trial. PSA, prostate-specific antigen; RT, radiotherapy.

RADICALS-RT recruited 1396 participants over 9 years between November 2007 and December 2016, 697 to the Adjuvant-RT Group and 699 to the Salvage-RT Policy Group (Figure 1). Median age was 65 years, median PSA at diagnosis was 7.9 ng/ml and 37% (517/1396) had a CAPRA-S score^18^ of 6+ (Table 1). Median PSA at randomisation was undetectable in both randomised groups. Median follow-up was 7.8 years at end of follow-up (December 2021).

### Treatment

**Figure 2:**
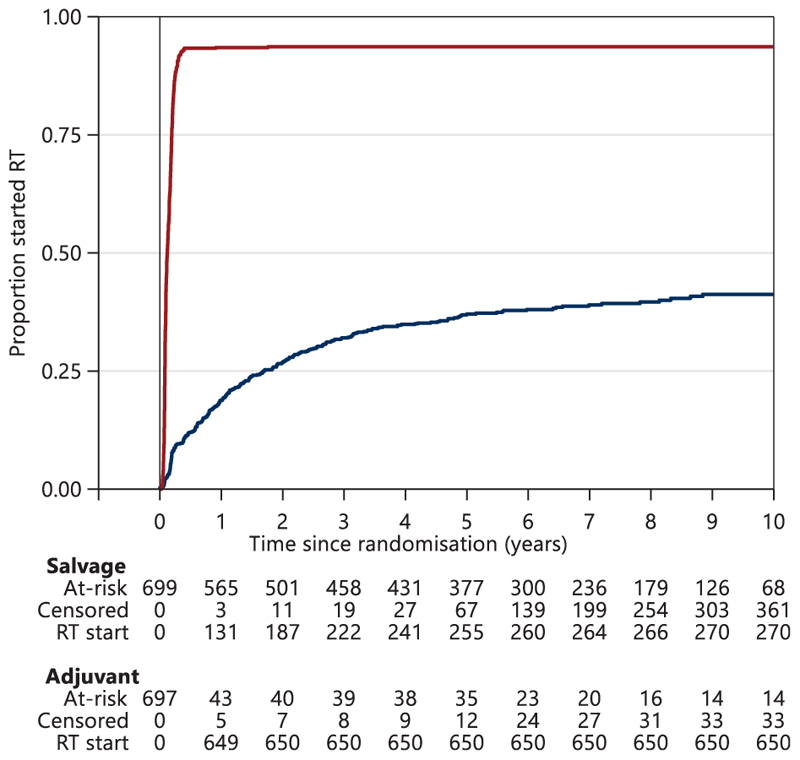
Proportion starting radiotherapy over time. Blue, Salvage-RT Policy Group; Red, Adjuvant-RT Group; RT, radiotherapy.

Most participants allocated to the Adjuvant-RT Group began treatment, as planned, shortly after randomisation (Figure 2). Ninety-three percent (648/697) in the Adjuvant-RT Group reported starting RT within 6 months at a median of 4.9 months [interquartile range (IQR) 4.1-5.6 months] after prostatectomy. At the time of analysis, 39% (270/699) of the Salvage-RT Policy Group had now reported starting salvage RT following PSA failure. In these 270 participants, the median time from randomisation to starting salvage RT was 1.5 years and their median PSA level at the time of starting salvage RT was 0.2 ng/ml (IQR 0.1-0.3 ng/ml). A further 12% (82/699) met the protocol definition of PSA failure during follow-up, but had not reported starting salvage RT at the time of analysis; for these 82 patients, median time from randomisation to PSA failure was 5.2 years.

Most participants who had RT received 66G Gy/30f (567, 62%) or 52.5 Gy/20f (268, 29%), with similar proportions in both randomised groups. Most participants received RT only to the prostate bed, with RT additionally to pelvic lymph nodes in only 3% (21/650) of the Salvage-RT Policy Group and 6% (17/270) of the Adjuvant-RT Group.

Among participants who reported starting RT, 156/650 (24%) of the Adjuvant-RT Group and 72/270 (27%) of the Salvage-RT Policy Group reported use of (neo-) adjuvant hormone therapy, either through co-enrolment in RADICALS-HD or as part of local standard of care.

### Primary outcome measure—freedom-from-distant-metastasis

**Figure 3:**
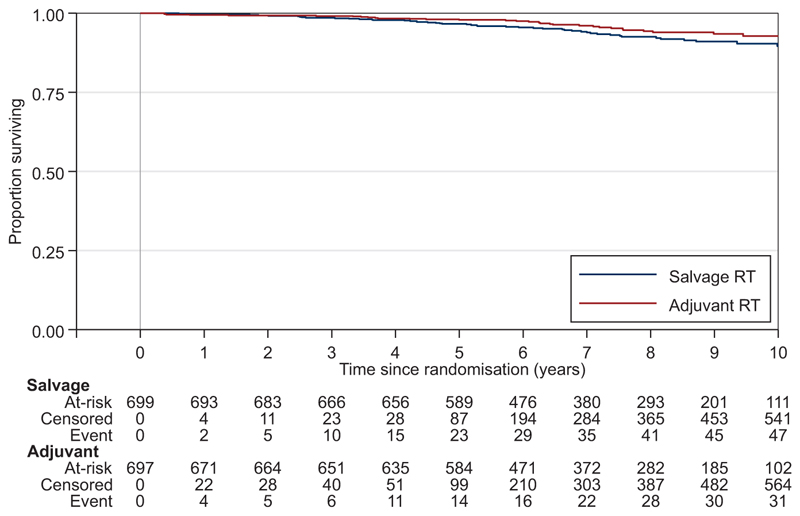
Freedom-from-distant-metastasis. Blue, Salvage-RT Policy Group; Red, Adjuvant-RT Group; RT, radiotherapy.

A primary outcome measure event of distant metastasis or death due to prostate cancer had been reported for 6% (80/1396) of participants at the end of follow-up, with 32 events in the Adjuvant-RT Group and 48 in the Salvage-RT Policy Group (Figure 3, Table 2). Of the 48 FFDM events in the Salvage-RT Policy Group, 37 followed after salvage RT, 7 followed PSA failure without reported salvage RT and 4 occurred in the absence of reported PSA failure. Sixty-three participants (28 in the Adjuvant-RT Group, 35 in the Salvage-RT Policy Group) reported distant metastasis but remained alive at the end of follow-up; 17 participants (4 in the Adjuvant-RT Group, 13 in the Salvage-RT Policy Group) reported metastasis followed by death due to prostate cancer. The difference between randomised groups was not statistically significant [HR=0.681, 95% confidence interval (CI) 0.432-1.072, *P*=0.095] for the Adjuvant-RT Group. There was no evidence of non-proportional hazards, *P*=0.695. The sensitivity analysis including suspicious, confirmed metastases with 8 additional events (1 Salvage-RT, 7 Adjuvant-RT) also showed no clear evidence of improvement in FFDM (Supplementary Table S1). Exploratory analyses of consistency of treatment effect on FFDM are depicted in Supplementary Figure S1, available at https://doi.org/10.1016/j.annonc.2024.03.010.

### Secondary outcome measures

**Figure 4:**
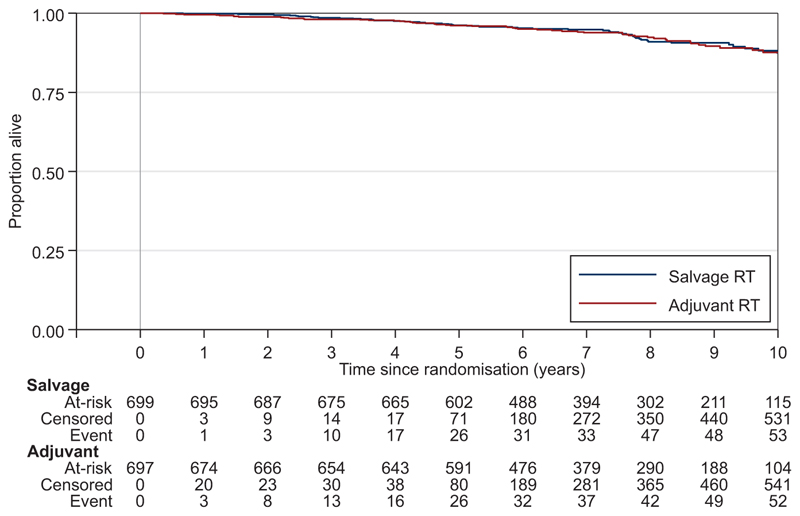
Overall survival. Blue, Salvage-RT Policy Group; Red, Adjuvant-RT Group; RT, radiotherapy.

When trial follow-up was stopped, overall, 109/1396 of the participants had died, with 52 deaths in the Adjuvant-RT Group and 57 deaths in the Salvage-RT Policy Group (Figure 4, Table 2). The difference between groups was not statistically significant, HR=0.980 for adjuvant treatment (95% CI 0.667-1.440, *P*=0.917), and there was no evidence of non-proportional hazards, *P*=0.322. Only 17 deaths were directly attributed to prostate cancer—4 in the Adjuvant-RT Group and 13 in the Salvage-RT Policy Group (Supplementary Figure S2, available at https://doi.org/10.1016/j.annonc.2024.03.010): HR=0.330 for Adjuvant-RT (95% CI 0.107-1.023, *P*=0.044).

**Figure 5:**
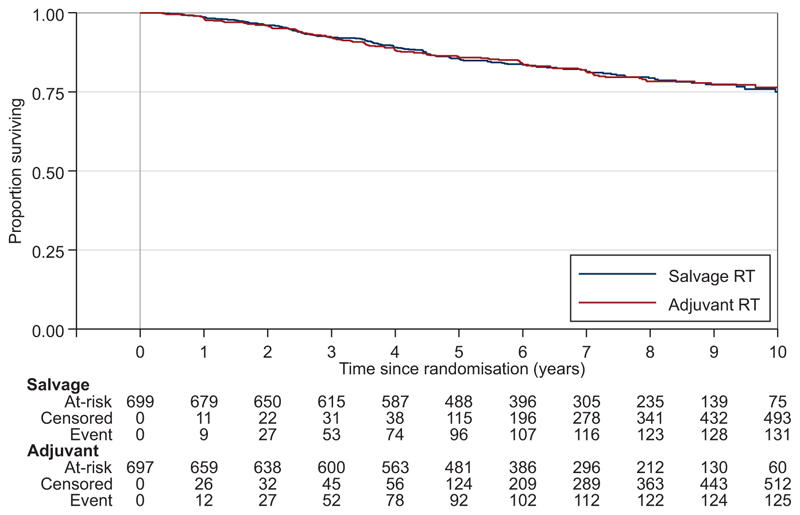
Biochemical progression-free survival. Blue, Salvage-RT Policy Group; Red, Adjuvant-RT Group; RT, radiotherapy.

We previously reported no difference in bPFS between randomised groups after a median 4.9 years of follow-up. Here, with a median 7.8 years of follow-up and 106 further events, there was still no evidence of a difference, HR=0.972 for Adjuvant-RT (95% CI 0.758-1.247, *P*=0.822) (Figure 5, Table 2).

**Figure 6:**
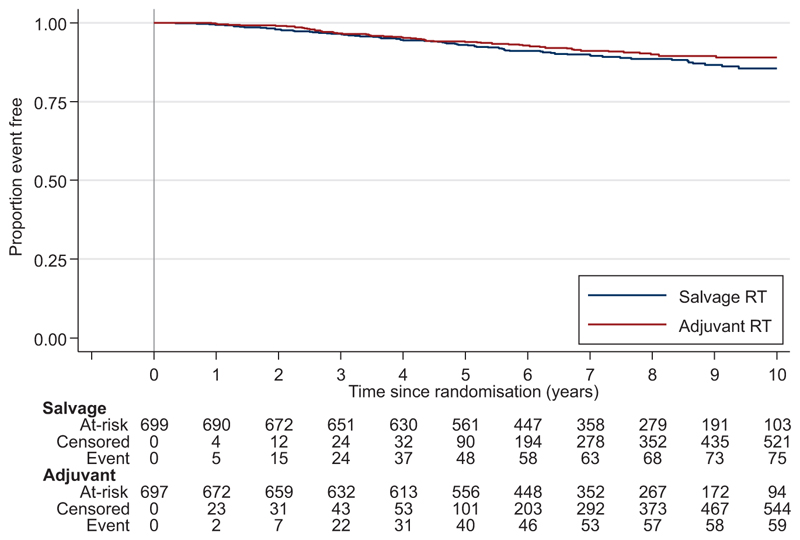
Initiation of non-protocol hormone therapy. Blue, Salvage-RT Policy Group; Red, Adjuvant-RT Group; RT, radiotherapy.

Non-protocol hormone therapy was initiated by 134 participants during follow-up, 59 in the Adjuvant-RT Group and 75 in the Salvage-RT Policy Group. The difference between groups was not statistically significant, HR = 0.832 for Adjuvant-RT (95% CI 0.589-1.176, *P*=0.297) (Figure 6, Table 2).

Grade 3 or 4 urethral stricture was reported for 81 participants (6%). Each of the other four routinely-recorded toxicities were reported at grade 3 or 4 for fewer than 5% of participants. Toxicity was more commonly-reported in the Adjuvant-RT Group, mainly a result of more grade 1 or 2 events, with late toxicity remaining significantly higher (Table 3).

**Figure 7:**
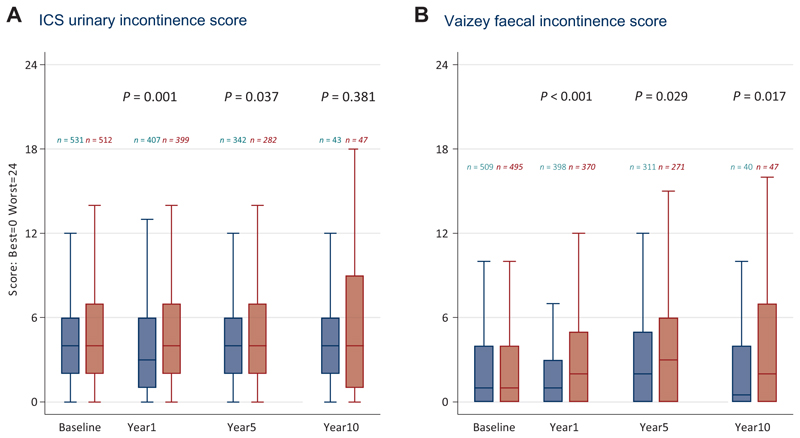
Incontinence ratings. Blue, Salvage-RT Policy Group; Red, Adjuvant-RT Group.

From patient-reported outcome measures (Figure 7), the Adjuvant-RT Group reported significantly worse incontinence 1 year after randomisation (*P*=0.001), but the evidence of difference lessened at later points. Faecal incontinence was statistically significantly worse after 1 year in the Adjuvant-RT Group (*P*<0.001), and was also statistically significantly different in participants with an assessment at 10 years after randomisation (*P*=0.017).

## Discussion

These long-term results from the RADICALS-RT trial have not shown any statistically significant or clinically meaningful benefit for adjuvant RT after radical prostatectomy in terms of FFDM. These findings are consistent with those previously reported on the early outcome measure, bPFS.^9^ The results confirm that adjuvant RT increases the risk of urinary and bowel morbidity. These data strengthen the case for observation after radical prostatectomy, keeping salvage RT in reserve in the event of recurrent disease.

Most of the secondary efficacy outcomes measures did not show any clear benefit for adjuvant RT: bPFS, time to non-protocol hormone therapy and overall survival were similar in the two arms of the trial. The prostate cancer-specific mortality (PCSM) result, which may appear intriguing, should be interpreted with considerable caution, given that it is based on only 17 events. Furthermore, it seems implausible that adjuvant RT should improve PCSM without a substantial effect on FFDM or time to non-protocol hormone therapy or both.

RADICALS-RT is the first randomised controlled trial that has both compared adjuvant versus early salvage RT and that is also sufficiently large and mature to report on FFDM. The two most mature randomised controlled trials, SWOG 8794 and EORTC 22911, did not include early salvage RT in the control arm, and are therefore of limited relevance to contemporary practice.^3,5^ Of the five randomised controlled trials that have compared adjuvant versus early salvage RT, the other four are not powered to study long-term outcomes such as FFDM. RADICALS-RT, which is the largest randomised controlled trial of adjuvant RT after radical prostatectomy, provides the best available evidence regarding the long-term effect of adjuvant RT on disease control.

RADICALS-RT has several strengths. The patient population, recruited primarily from the UK, Denmark and Canada, is representative of men undergoing radical prostatectomy internationally. The rate of PSA failure after radical prostatectomy alone was relatively high, at around 50%, and therefore suitable for a trial testing the impact of adjuvant RT. Compliance with allocated treatment and follow-up was high and was consistent across both arms. Outcome measures included not only physician-assessed toxicity, but also patient-reported functional outcomes. The use of (neo-) adjuvant hormone therapy with RT was left to local choice to reflect the breadth of practice at trial initiation, with co-enrolment in RADICALS-HD encouraged. Around one-quarter of participants reported having (neo-) adjuvant hormone therapy with their RT. While proportionately similar, only around half of participants in the Salvage-RT Policy Group were exposed to RT, so the absolute number of participants having hormone therapy with RT was greater in the Adjuvant-RT Group. This may have implications for interpreting the non-protocol hormone therapy data.

RADICALS-RT also has some limitations. Since RADICALS-RT opened, new evidence has suggested that men receiving post-operative RT benefit from the addition of hormone therapy.^16^ While greater use of hormone therapy may have improved outcomes, data from RADICALS-HD suggest that the benefit of hormone therapy is similar, regardless of RT timing.^13^ Similarly, results from the RTOG SPPORT trial^19^ suggest a benefit to treating not just the prostate bed, but also the pelvic lymph nodes in men receiving salvage RT. This option was permitted in RADICALS-RT, but over 95% of participants who had RT received it to the prostate bed alone. Once again, there is no evidence that pelvic nodal RT would have a differential effect in the adjuvant or salvage setting. Advances in treatment, such as these, provide another argument in favour of a salvage RT policy. Given that patients may receive salvage RT years after their prostatectomy, they may benefit from new knowledge not available in the immediate post-operative period.

The ARTISTIC meta-analysis collaboration was developed to include all the relevant randomised trials of post-operative RT timing, and, with continued follow-up of all trials, will be powered to report on FFDM and overall survival.^20^ The meta-analysis will also enable subgroup analyses to investigate whether any subgroup could be identified to benefit from adjuvant RT.

The long-term results from the RADICALS-RT trial have not shown sufficient benefit for adjuvant RT in comparison to a policy of salvage RT for PSA failure; but adjuvant RT does increase the risk of urinary and bowel morbidity. These findings add support to a policy of observation after radical prostatectomy, with salvage RT used in the event of PSA failure.

## Supplementary Material

Supp 1

## Figures and Tables

**Table 1:** Patient characteristics

	Salvage-RT				Adjuvant-RT				All		
	*n*		%		*n*		%		*N*		%
	699		(100)		697		(100)		1396		(100)
Age											
Years^a^	65 (60-68)				65 (60-68)				65 (60-68)		
PSA at diagnosis											
ng/ml^a^	8 (5.6-11.6)				7.8 (5.8-11.4)				7.9 (5.7-11.5)		
Gleason score											
GS < 7	48		(7)		48		(7)		96		(7)
GS 3 + 4	338		(48)		349		(50)		687		(49)
GS 4 + 3	190		(27)		188		(27)		378		(27)
GS ≥ 8	123		(18)		112		(16)		235		(17)
Pathologic T-stage											
pT2	176		(25)		163		(23)		339		(24)
pT3a	390		(56)		408		(59)		798		(57)
pT3b	129		(18)		121		(17)		250		(18)
pT4	4		(1)		5		(1)		9		(1)
Positive margins											
Present	444		(64)		439		(63)		883		(63)
Absent	255		(36)		258		(37)		513		(37)
Lymph node involvement											
N1	28		(4)		38		(5)		66		(5)
N0	374		(54)		336		(48)		710		(51)
Nx	297		(42)		322		(46)		619		(44)
Missing	0				1				1		
CAPRA-S score											
Low (0-2)	55		(8)		58		(8)		113		(8)
Intermediate (3-5)	384		(55)		382		(55)		766		(55)
High (6+)	260		(37)		257		(37)		517		(37)
Country											
England	573		(82)		574		(82)		1147		(82)
Denmark	92		(13)		95		(14)		187		(13)
Canada	28		(4)		22		(3)		50		(4)
Republic of Ireland	6		(1)		6		(1)		12		(1)

*n* (%) unless indicated.

CAPRA, Cancer of the Prostate Risk Assessment; GS, score; IQR, interquartile range; N, nodal status; PSA, prostate-specific antigen; RT, radiotherapy; T, tumour stage.

a Median (IQR).

**Table 2:** Primary and secondary outcome measures

	Salvage-RT			Adjuvant-RT	
	(*n* = 699)			(*n* = 697)	
Freedom-from-distant-metastasis					
Events	48	(6.9%)		32	(4.6%)
Metastasis, no PCa death	35			28	
Prostate cancer death	13			4	
Hazard ratio^a^	–			0.681 (0.432-1.072)	
Log-rank *P* value^a^				0.095	
Proportional hazards *P* value^b^				0.695	
RMST^c^ (95% CI)	9.61	(9.49-9.72)		9.72	(9.62-9.82)
10-year event-free for FFDM	89.6%			92.7%	
Overall survival					
Events	57	(8.2%)		52	(7.5%)
Hazard ratio^a^	–			0.980 (0.667-1.440)	
Log-rank *P* value^a^				0.917	
Proportional hazards *P* value^b^				0.322	
RMST^c^ (95% CI)	9.58	(9.47-9.69)		9.56	(9.44-9.68)
10-year survival	87.4%			87.6%	
Prostate cancer-specific mortality					
Events	13			4	
Hazard ratio^a^	–			0.330 (0.107-1.023)	
Log-rank *P* value^a^				0.044	
Proportional hazards *P* value^b^				0.765	
RMST^c^ (95% CI)	9.90	(9.85-9.96)		9.97	(9.94-10.0)
10-year event-free for PCSM	97.1%			99.2%	
Initiation of non-protocol HT					
Events	75	(10.7%)		59	(8.5%)
Hazard ratio^a^	–			0.832 (0.589-1.176)	
Log-rank *P* value^a^				0.297	
Proportional hazards *P* value^b^				0.854	
RMST^c^ (95% CI)	9.30	(9.15-9.46)		9.43	(9.29-9.57)
10-year event-free for FFDM	85.4%			88.9%	
Biochemical PFS					
Events	135	(19.3%)		125	(17.9%)
Hazard ratio^a^	–			0.972 (0.758-1.247)	
Log-rank *P* value^a^				0.822	
Proportional hazards *P* value^b^				0.527	
RMST^c^ (95% CI)	8.70	(8.50-8.90)		8.72	(8.51-8.93)
10-year event-free for bPFS	75.0%			76.4%	

CI, confidence interval; FFDM, freedom-from-distant-metastasis; HR, hazard ratio; HT, hormone therapy; PCa, prostate cancer; PFS, progression-free survival; RMST, restricted-mean ‘survival’ time; RT, radiotherapy.

a Adjusted for randomisation stratification factors.

b Grambsche–Therneau test of non-proportional hazards.

c Restricted mean survival time (standard error).

**Table 3:** RTOG toxicity scale^a^

	Early (<2 years)							Late (2 + years)						
	All		Salvage-RT		Adjuvant-RT		*P* ^b^	All		Salvage-RT		Adjuvant-RT		*P* ^b^
	*N*	%	*n*	%	*n*	%		*N*	%	*n*	%	*n*	%	
	1379	(100)	697	(100)	682	(100)		1343	(100)	681	(100)	662	(100)	
Diarrhoea														
Grade 1 or 2	398	(29)	127	(18)	271	(40)	<0.001	184	(14)	64	(9)	120	(18)	<0.001
Grade 3	16	(1)	4	(1)	12	(2)		7	(1)	2	(<1)	5	(1)	
Grade 4	0	(0)	0	(0)	0	(0)		1	(<1)	0	(0)	1	(<1)	
Proctitis														
Grade 1 or 2	216	(16)	52	(7)	164	(24)	<0.001	130	(10)	43	(6)	87	(13)	<0.001
Grade 3	11	(1)	3	(<1)	8	(1)		10	(1)	2	(<1)	8	(1)	
Grade 4	0	(0)	0	(0)	0	(0)		0	(0)	0	(0)	0	(0)	
Cystitis														
Grade 1 or 2	284	(21)	96	(14)	188	(28)	<0.001	141	(11)	50	(7)	91	(14)	0.001
Grade 3	20	(1)	6	(1)	14	(2)		14	(1)	7	(1)	7	(1)	
Grade 4	1	(<1)	0	(0)	1	(<1)		0	(0)	0	(0)	0	(0)	
Haematuria														
Grade 1 or 2	130	(9)	37	(5)	93	(14)	<0.001	129	(10)	31	(5)	98	(15)	<0.001
Grade 3	29	(2)	5	(1)	24	(4)		35	(3)	5	(1)	30	(5)	
Grade 4	0	(0)	0	(0)	0	(0)		0	(0)	0	(0)	0	(0)	
Urethral stricture														
Grade 1 or 2	73	(5)	22	(3)	51	(8)	0.001	66	(5)	22	(3)	44	(7)	<0.001
Grade 3	76	(6)	32	(5)	44	(6)		55	(4)	19	(3)	36	(5)	
Grade 4	5	(<1)	3	(<1)	2	(<1)		3	(<1)	3	(<1)	0	(0)	

*n* (%) unless indicated.

RT, radiotherapy; RTOG, Radiation Therapy Oncology Group.

a No grade 5 events reported.

b Adjuvant versus Salvage, chi-square test.

## Data Availability

All of the information is publicly available. The dataset and technical appendices are available upon request as per the controlled access approach of the MRC CTU at UCL. Please contact the corresponding author for more information.
