# Supplementary material for: Timing of radiotherapy (RT) after radical prostatectomy (RP): long-term outcomes in the RADICALS-RT trial (NCT00541047)
Source: Ann Oncol. Author manuscript; Available in PMC 2024 Dec 10. (PMC7617161; doi:10.1016/j.annonc.2024.03.010)
Supplement: Supp 1 [file EMS197974-supplement-Supp_1.zip › 1-s2.0-S0923753424001054-mmc1.docx]

**Supplementary Table 1: Sensitivity analysis of primary outcome – includes suspicious unconfirmed metastases as events**

|  | **Salvage‑RT** | | **Adjuvant‑RT** | |
| --- | --- | --- | --- | --- |
|  | **(n=699)** | | **(n=697)** | |
| **Freedom-from-distant-metastasis** |  |  |  |  |
| Events | 49 | (7.0%) | 39 | (5.6%) |
| Metastasis, no PCa death | 36 |  | 35 |  |
| Prostate cancer death | 13 |  | 4 |  |
| Hazard ratio* |  | | HR = 0.830 (0.541, 1.272) | |
| Log-rank p * |  |  | 0.392 |  |
| Proportional hazards p** |  |  | 0.777 |  |
| RMST^†^ (SE) | 9.60 | (9.48, 9.71) | 9.65 | (9.53, 9.76) |
| 10-year event free for MFS | 89.1% |  | 91.6% |  |

* adjusted for randomisation stratification factors

** Grambsch-Therneau test of non-proportional hazards

† Restricted mean survival time (standard error)

PCa = prostate cancer
