# Supplementary figures and images for: Timing of radiotherapy (RT) after radical prostatectomy (RP): long-term outcomes in the RADICALS-RT trial (NCT00541047)
Source: Ann Oncol. Author manuscript; Available in PMC 2024 Dec 10. (PMC7617161; doi:10.1016/j.annonc.2024.03.010)

## Slide 1
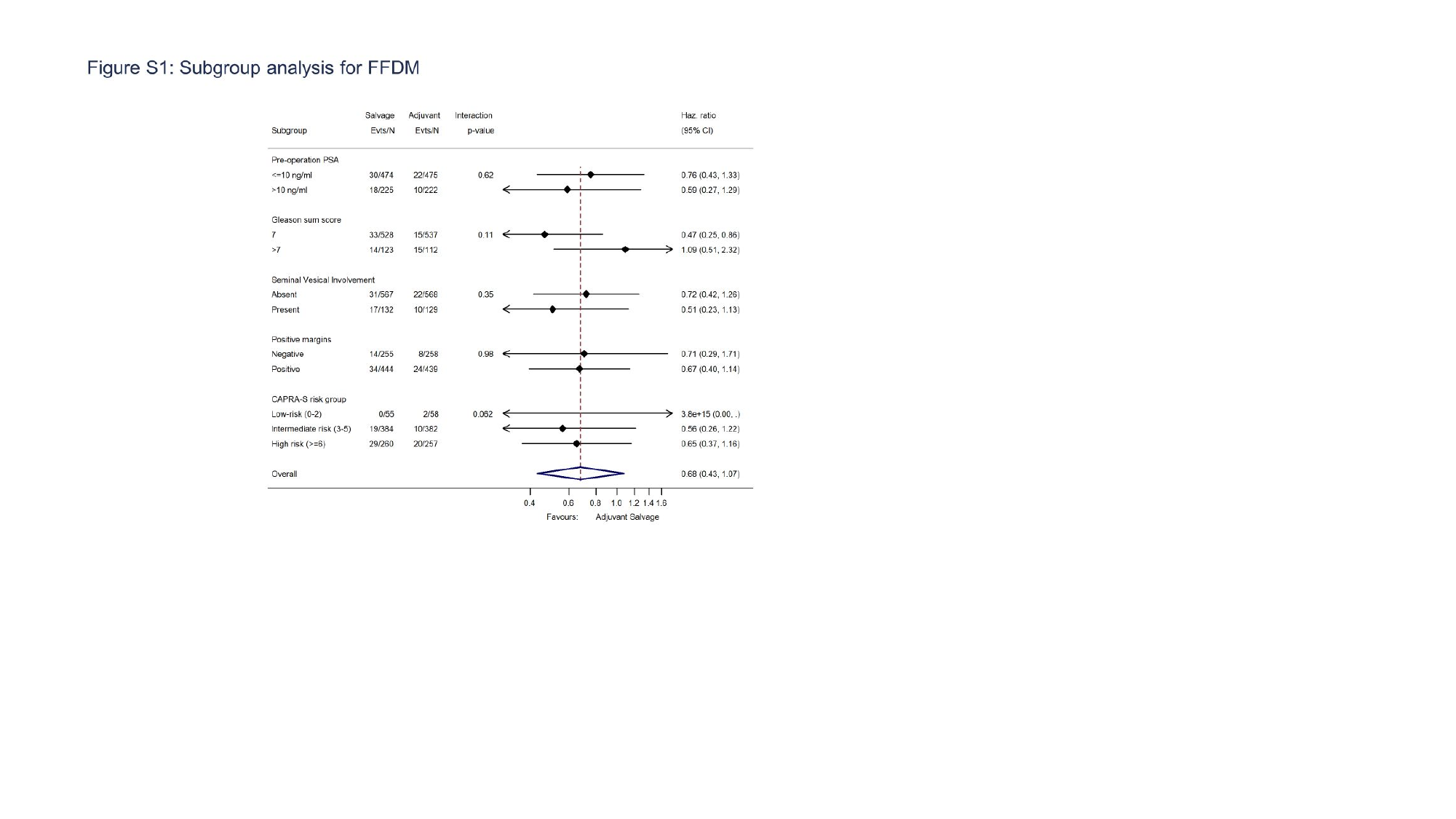

## Slide 2
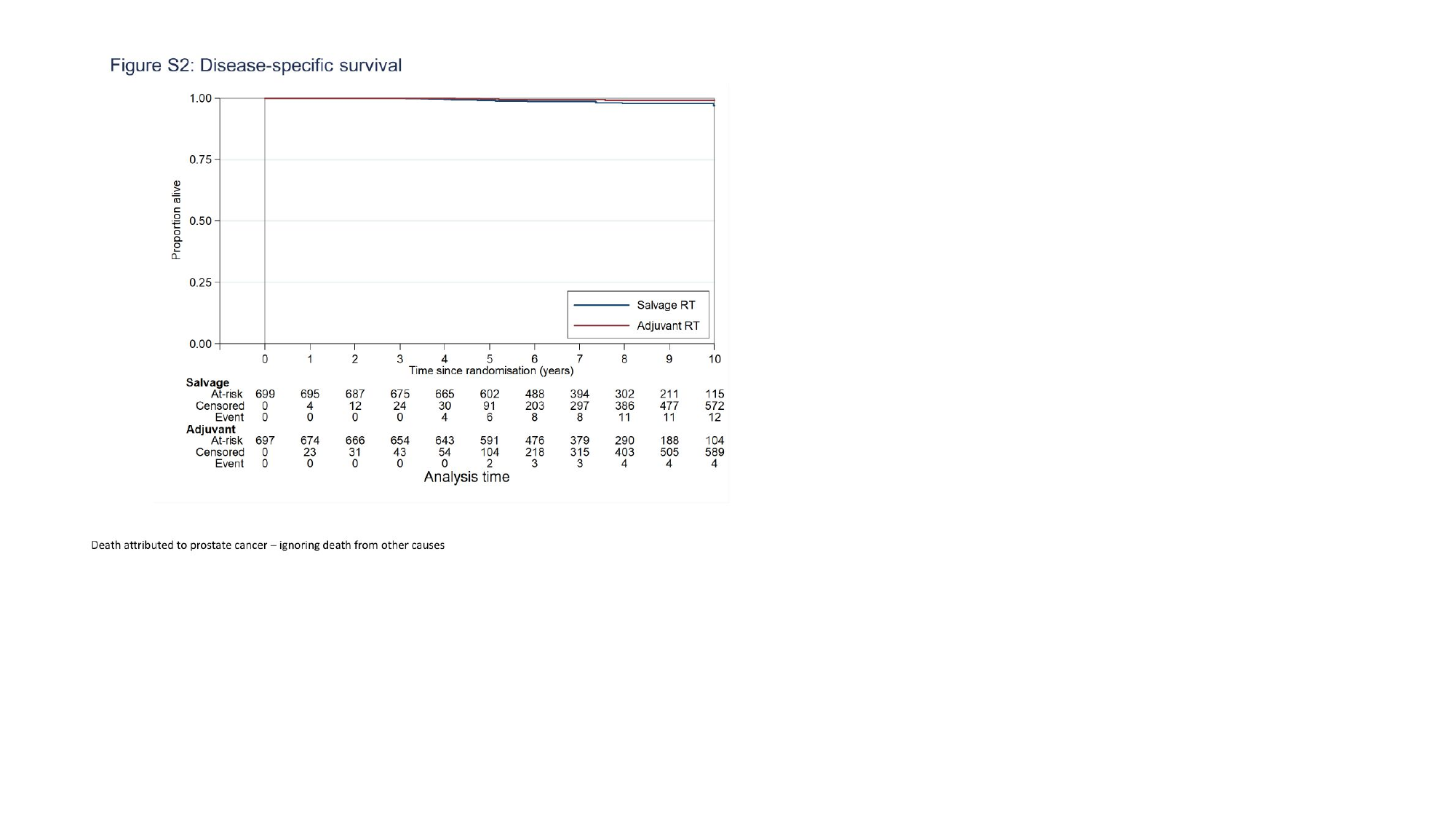

Supplement: Supp 1 [file EMS197974-supplement-Supp_1.zip › 1-s2.0-S0923753424001054-mmc2.pptx]
